# Institutional support, relationships, and multicultural participation: response surfaces of university belonging and sociocultural engagement

**DOI:** 10.3389/fpsyg.2026.1950905

**Published:** 2026-10-16

**Authors:** Siyuan Luo, Hui Qu

**Affiliations:** 1 School of Liberal Arts Education and Art Media, Xiamen Institute of Technology, Xiamen, China; 2 Lixin School of Accounting, Beijing College of Finance and Commerce, Beijing, China

**Keywords:** campus environment, cultural psychology, multicultural participation, open data, relationship quality, response surface analysis, sociocultural engagement, university belonging

## Abstract

**Introduction:**

Universities are cultural settings in which institutional arrangements and interpersonal recognition jointly condition participation and belonging.

**Methods:**

This open-data secondary analysis examined how perceived supportive campus environment and relationship quality combined in relation to university belonging and sociocultural engagement among health-professions students at a culturally diverse university in the United Arab Emirates. The released dataset contained 683 records; 661 complete cases entered both polynomial models. Predictors were transformed to proportion-of-maximum-possible scores. Inference combined 2,000 bootstrap resamples, HC3 heteroskedasticity-robust standard errors, duplicate-record checks, and Cook's D influence sensitivity.

**Results:**

The models explained 47.3% of belonging and 25.6% of sociocultural engagement. The joint-resource slope was positive for belonging [a1 = 3.159, 95% bootstrap CI (2.596, 3.602)] and sociocultural engagement [a1 = 1.721, CI (1.233, 2.133)]. Curvature, directional difference, and mismatch curvature were not consistently supported. Removing two exact duplicate records or 41 observations above Cook's *D*>4/*n* retained the positive joint-resource pattern.

**Discussion:**

Thus, stronger institutional support and relationships were jointly associated with both campus belonging and multicultural participation, without evidence for a unique matching effect. The cross-sectional measures do not directly observe identity co-construction, so the contribution is a bounded quantitative account of conditions associated with culturally situated participation and belonging.

## 1 Introduction

Globalized universities bring students into contact with institutional expectations, professional communities, and peers from diverse social and cultural backgrounds. These settings do more than provide instruction. They organize recurring encounters through which students learn what forms of participation are valued, whose contributions are recognized, and whether they can see themselves as legitimate members of an academic community. Cultural psychology describes persons and cultural worlds as mutually constituted: individuals act within socially organized environments, while those environments acquire meaning through participation and interpretation. Contemporary cross-cultural research likewise shows that selfhood cannot be reduced to a simple East-West dichotomy; cultural models of self vary across dimensions, relationships, and local contexts (Vignoles et al., 2016).

University belonging is an especially useful construct for examining this relational interface. It refers to feeling accepted, valued, included, and connected to an institution and its members (Freeman et al., 2007; Hoffman et al., 2002; Strayhorn, 2018). Belonging has been associated with engagement, persistence, achievement, wellbeing, and reduced burnout (Aker and Şahin, 2022; Allen et al., 2024; Gillen-O'Neel, 2021). A recent systematic review and meta-analysis likewise found positive associations between university belonging and both academic and wellbeing outcomes, while emphasizing substantial heterogeneity and the need for stronger causal designs (van Kessel et al., 2025). Yet belonging is not distributed solely by individual motivation or personality. Campus climate, inclusive practices, interpersonal recognition, and access to participation opportunities shape whether students experience membership as credible (Locks et al., 2008; Masika and Jones, 2016; Meeuwisse et al., 2010). Recent ecological and cultural-script accounts therefore urge researchers to locate belonging across interpersonal, institutional, and structural levels, including the power-laden practices through which universities communicate who is accepted, rather than treating belonging as a decontextualized student trait (Covarrubias, 2024; Johnson, 2026).

This relational framing is particularly relevant in health-professions education. Students enter demanding programs in which learning depends on collaboration, feedback, psychological safety, and gradual participation in professional communities. Positive peer and faculty relationships support engagement and can reduce isolation and burnout (Baños et al., 2019; Geraghty et al., 2020; Tsuei et al., 2019; Ziegelstein, 2018). At the same time, institutional priorities determine whether students have access to academic support, cross-cultural interaction, social activities, wellbeing services, and spaces for discussing consequential social issues. These resources are not merely background amenities. They can communicate who is expected to participate and what kinds of membership the university makes possible.

Classic belonging theory emphasizes a broad human need for stable, positive relationships (Baumeister and Leary, 1995). Higher-education scholarship extends this account to institutional membership, distinguishing classroom belonging, peer connectedness, faculty support, and identification with the university (Freeman et al., 2007; Strayhorn, 2018). Systematic evidence indicates that students describe belonging through connectedness, safety, acceptance, value, and inclusion, and that these experiences are responsive to university-wide practices as well as local relationships (Allen et al., 2024). Research with ethnically diverse groups further shows that the learning environment and interaction quality jointly contribute to belonging and academic success (Meeuwisse et al., 2010). Broader engagement models similarly locate outcomes at educational interfaces (Kahu, 2013; Kahu and Nelson, 2018; Trowler et al., 2022; Tian et al., 2021).

Ecological and intersectional perspectives deepen the argument by asking how identities, power, and institutional structures condition these experiences. The same classroom, advising encounter, or campus policy can communicate inclusion to some students and marginality to others (Johnson, 2026). This does not mean that every study of belonging directly measures identity negotiation. Rather, belonging can be treated as a bounded indicator of person-context relations: a judgment that one's presence is legitimate, one's participation is recognized, and the institution is a plausible component of the self. In culturally diverse universities, such judgments are embedded in interactions across nationality, language, professional background, gender, and social position, even when a dataset cannot separately estimate each intersection.

Supportive campus environments operate through multiple channels. Academic and learning services can reduce barriers to participation; campus activities can create shared practices; wellbeing services can signal institutional care; and structured engagement across diverse backgrounds can build intergroup competence (Denson and Bowman, 2013; Denson and Zhang, 2010; Locks et al., 2008). In health-professions education, inclusive and culturally responsive learning environments are additionally important because students are preparing for practice in diverse communities (Davis et al., 2021; Kassab et al., 2023).

Relationship quality provides the interpersonal counterpart. Interactions with peers, faculty, advisors, and staff make institutional values concrete. Respectful relationships can support psychological safety, feedback seeking, and agentic engagement; negative or exclusionary interactions can make formal support inaccessible in practice (Geraghty et al., 2020; Raufelder et al., 2015; Tsuei et al., 2019). Communities-of-practice theory connects these levels by conceptualizing identity as negotiated participation in a social learning system (Wenger, 1998, 2010). Institutional arrangements create the boundaries and opportunities of participation, while relationships shape whether newcomers are recognized as members.

Kassab et al. (2024a) brought these constructs together in a path model of health-professions students. They found that supportive campus environment and relationship quality were positively associated with belonging and sociocultural engagement. That analysis established net linear associations and mediation paths. It did not, however, address how the two resources combine. Additive coefficients alone cannot show whether high support and high relationship quality accumulate, whether one resource compensates for the other, whether imbalance carries a penalty, or whether the association becomes nonlinear at higher levels.

Difference scores are an intuitive but statistically ambiguous way to represent alignment between two resources because the same difference can arise from very different component levels. Polynomial regression retains the two predictors, their squared terms, and their interaction, allowing the fitted surface to be interpreted along theoretically meaningful lines (Edwards and Parry, 1993). RSA is therefore useful not only for classical person-environment fit questions but also for examining whether two contextual resources jointly accumulate, curve, or diverge. At the same time, methodological guidance warns against calling any positive congruence-line slope a “congruence effect.” A genuine matching claim requires a broader pattern of surface conditions; uncertainty around curvature and incongruence parameters must be considered (Humberg et al., 2019; Yao and Ma, 2023).

The present study uses response surface analysis (RSA) to extend rather than reproduce the original path analysis. It asks: (RQ1) How are supportive campus environment and relationship quality jointly associated with university belonging and sociocultural engagement? (RQ2) Do the outcomes increase when both contextual resources rise together, or do their surfaces show curvature? (RQ3) Is imbalance between institutional support and relationship quality associated with either outcome?

The need for this study is timely and practical. Universities are serving increasingly culturally diverse student bodies at the same time that belonging is linked to persistence, wellbeing, and academic outcomes (Allen et al., 2024; van Kessel et al., 2025). Institutions often invest separately in services, inclusion initiatives, or relationship-building, yet additive analyses do not tell decision makers whether one layer can compensate for another or whether the strongest outcomes occur when both are favorable. The study changes what is known in four ways. First, it treats university belonging as a bounded relational outcome jointly situated in institutional conditions and everyday relationships, rather than as an isolated student attitude. Second, it adds sociocultural engagement—eight cross-cultural interaction and five adaptation items—to test whether the same joint pattern extends from feeling accepted to participating across cultural difference. Third, RSA distinguishes the practically important joint resource gradient from unsupported claims about matching, imbalance, or an optimal equality point. Fourth, the study provides an auditable open-data workflow with HC3 inference and duplicate- and influence-point sensitivity. The unique contribution is therefore not merely another positive association: it shows that the most defensible pattern is cumulative strength across both resource layers, while ruling out stronger congruence interpretations. These contributions do not establish longitudinal identity transformation, causal co-construction, or effects specific to international students. Figure 1 summarizes the bounded conceptual model.

**Figure 1:**
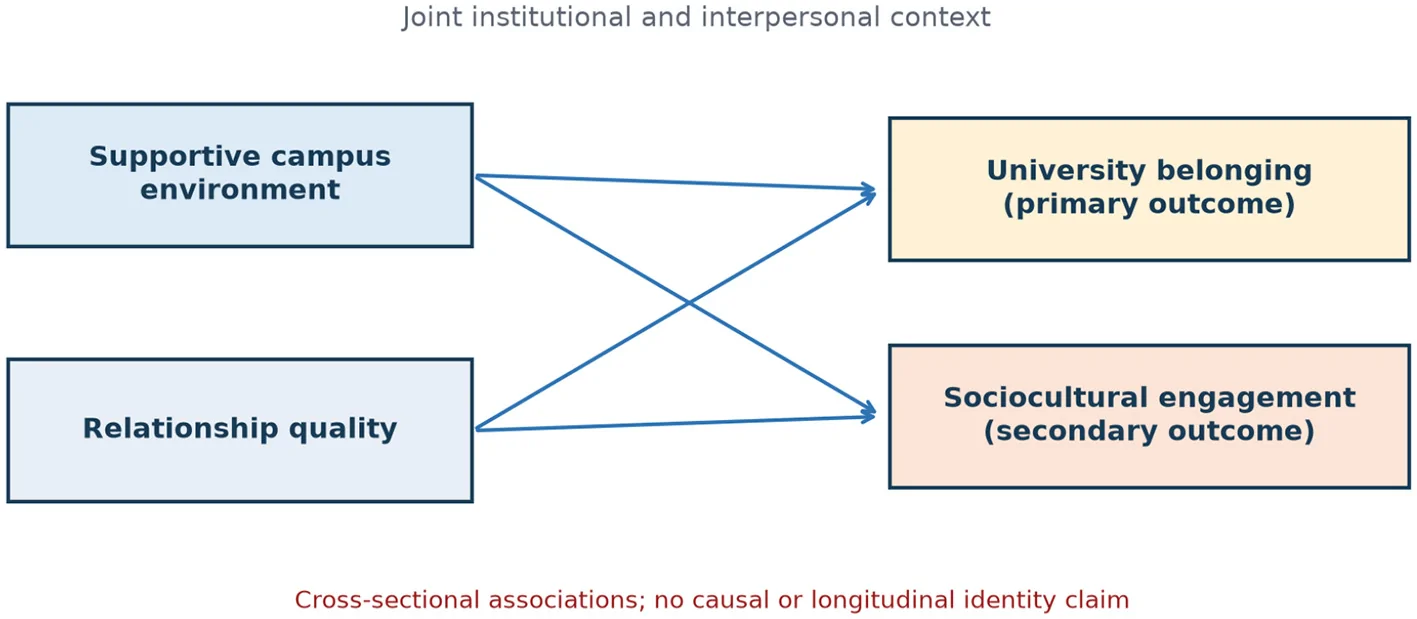
Conceptual model of institutional and interpersonal resources as joint correlates of belonging and sociocultural engagement.

## 2 Materials and methods

### 2.1 Study design and context

This study was a cross-sectional, quantitative secondary analysis of openly released survey data. The original study was conducted at Gulf Medical University in Ajman, United Arab Emirates, across medicine, dentistry, pharmacy, nursing, and health-sciences programs. Students were recruited between 25 October and 14 November 2023 using convenience sampling and completed an anonymous online questionnaire during class. The institution reported a student body representing 77 nationalities, making it a culturally diverse setting even though the released data do not identify all students as international or support country-specific inference (Kassab et al., 2024a).

The original research protocol was approved by the Gulf Medical University Research Ethics Committee (IRB-COM-FAC-16-SEPT-2023), and participants provided written informed consent. The present analysis used anonymized public data and introduced no new participant contact. The dataset was obtained from Mendeley Data (Version 1; Kassab et al., 2024b) under a CC BY 4.0 license.

### 2.2 Provenance audit and analytic sample

The Version 1 repository record and released SPSS file contain 683 student records and 48 variables (Kassab et al., 2024b), whereas the linked article reports *n* = 638 (Kassab et al., 2024a). The released file was defined prospectively for this reanalysis as the analysis object. Accordingly, all descriptive results use the 683 released records and both polynomial models use the 661 records with complete focal outcomes, age, and gender. Specifically, all four focal composites were available for 683 records, age for 664, and gender for 680; complete-case intersection across the focal variables and both covariates yielded 661. Neither the repository metadata nor the released file contains an exclusion flag or documented rule that recovers 638 cases. We therefore did not force the public file to match the article and do not speculate about the cause of the discrepancy. Reproducibility in this article is exact with respect to the released Version 1 file, while reconciliation with the source article remains dependent on clarification from the data creators.

Two records were exact duplicates across all released variables. They were retained in the primary analysis because the public file contains no identifier or audit trail that establishes which occurrence, if any, is erroneous. Sensitivity analyses removed the two extra duplicate records (*n* = 659) and, separately, observations with Cook's *D*>4/*n* in the primary belonging model (*n* = 620).

### 2.3 Measures and construct alignment

The eight supportive-environment items covered academic support, learning services, cross-background contact, social involvement, wellbeing, nonacademic responsibilities, campus participation, and engagement with social or political issues. Four relationship items assessed perceived relationship quality with peers, faculty, academic advisors, and administrative staff. Five belonging items assessed fit, welcome, pride, positive attachment, and the university as a favored place (Freeman et al., 2007; Kassab et al., 2024a; Mendoza-Denton et al., 2002). The secondary outcome, sociocultural engagement, was the exact mean of the 13 items used in the released composite: eight cross-cultural interaction items and five sociocultural adaptation items. It measures multicultural participation, not identity negotiation itself. All four item-based composites were complete for the 683 released records. Cronbach's alpha was 0.926 for supportive environment, 0.922 for relationship quality, 0.922 for belonging, and 0.956 for sociocultural engagement. Table 1 summarizes construct alignment (Boateng et al., 2018).

**Table 1:** Construct alignment and operationalization of study variables.

Construct	Operationalization	Range	Role in this study
Supportive campus environment	Mean of IS1–IS8	1–4	Institutional/contextual resource
Relationship quality	Mean of IPI1–IPI4	1–5	Interpersonal resource
University belonging	Mean of SB1–SB5	1–5	Primary relational outcome
Sociocultural engagement	Mean of 13 SCE items	1–4	Secondary multicultural-participation outcome
Age	Released age in years; standardized	15–53	Covariate
Gender	Released binary code; centered	1–2	Covariate; not an identity analysis

### 2.4 Statistical model and surface parameters

The predictors used different response ranges, so both composites were transformed to proportion-of-maximum-possible scores: Xraw = (support – 1)/3 and Yraw = (relationships – 1)/4. Each was then centered at 0.50, yielding *X* = Xraw – 0.50 and *Y* = Yraw – 0.50. This places both resources on a comparable 0-to-1 metric without standardizing away their theoretical endpoints.

We estimated the following second-order polynomial regression (Equation 1):

$$\begin{array}{lll} B=b_{0} & + & b_{1}X+b_{2}Y+b_{3}X^{2}+b_{4}XY+b_{5}Y^{2}+b_{6}\text{Ag}{\text{e}}_{z} \end{array}$$

$$\begin{array}{ll} + & b_{7}\text{Gende}{\text{r}}_{c}+\epsilon . \end{array}$$ (1)

The response-surface parameters were *a*_1_ = *b*_1_+*b*_2_, representing the slope along *X* = *Y*; *a*_2_ = *b*_3_+*b*_4_+*b*_5_, representing curvature along *X* = *Y*; *a*_3_ = *b*_1_−*b*_2_, representing direction along *X* = −*Y*; and *a*_4_ = *b*_3_−*b*_4_+*b*_5_, representing curvature along *X* = −*Y*. Positive *a*_1_ indicates that the outcome rises as both predictors increase together, but does not by itself establish a unique congruence effect. Negative *a*_4_ would be consistent with a mismatch penalty only when supported by its interval and the broader surface conditions.

Separate ordinary least squares polynomial models were fitted for university belonging (primary outcome) and sociocultural engagement (topic-aligned secondary outcome) using the same complete-case sample. For coefficient-level inference we report HC3 heteroskedasticity-robust standard errors and *t* tests. Ninety-five percent percentile confidence intervals for *a*_1_–*a*_4_ were obtained from 2,000 nonparametric bootstrap resamples (seeds 2604011 and 2604012), and HC3 delta-method standard errors were also calculated for those linear combinations. Influence was evaluated using Cook's *D*>4/*n* in the primary belonging model; flagged observations were removed only for sensitivity analysis. Exact-duplicate removal was examined separately. Because the reanalysis followed public data release, it was not preregistered. The workflow is shown in Figure 2. The analysis code, source-data access instructions, and reference numerical outputs are provided in Supplementary Data Sheet 1.

**Figure 2:**
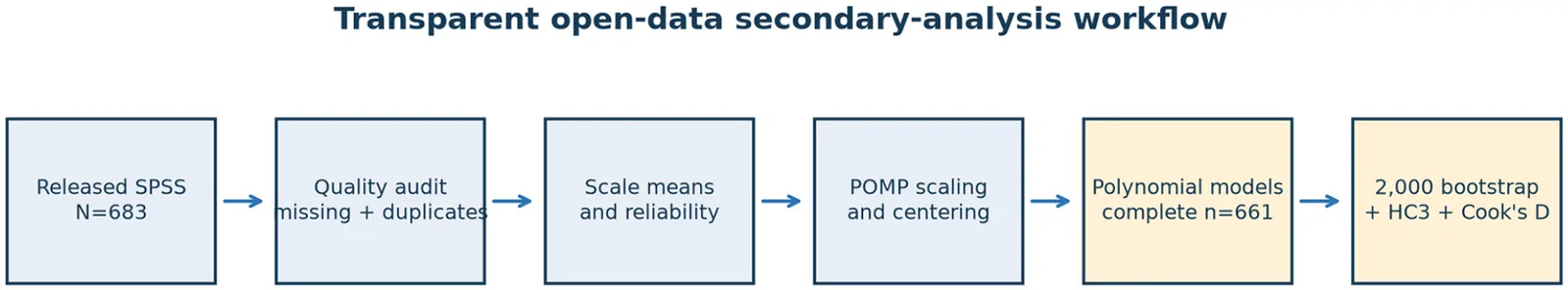
Transparent workflow from released data to bootstrap, HC3, and influence-point inference.

## 3 Results

### 3.1 Sample and score distributions

The released dataset contained 683 records; the two polynomial models used 661 complete cases. Gender was recorded for 680 students and age for 664. Mean age was 20.28 years (SD = 4.49). Mean scores were 3.007 (SD = 0.751) for supportive environment, 3.802 (SD = 0.820) for relationship quality, 3.725 (SD = 0.834) for belonging, and 3.156 (SD = 0.680) for sociocultural engagement. Observations were concentrated toward the upper half of the scales, so predictions near the lowest joint-resource levels are less data-dense. See Table 2 and Figure 3.

**Table 2:** Descriptive statistics and scale reliability.

Variable	*n*	Mean	SD	Observed range	α
Supportive campus environment	683	3.007	0.751	1.00–4.00	0.926
Relationship quality	683	3.802	0.820	1.00–5.00	0.922
University belonging	683	3.725	0.834	1.00–5.00	0.922
Sociocultural engagement	683	3.156	0.680	1.00–4.00	0.956
Age	664	20.282	4.490	15.00–53.00	–
Gender code	680	1.269	0.444	1.00–2.00	–

**Figure 3:**
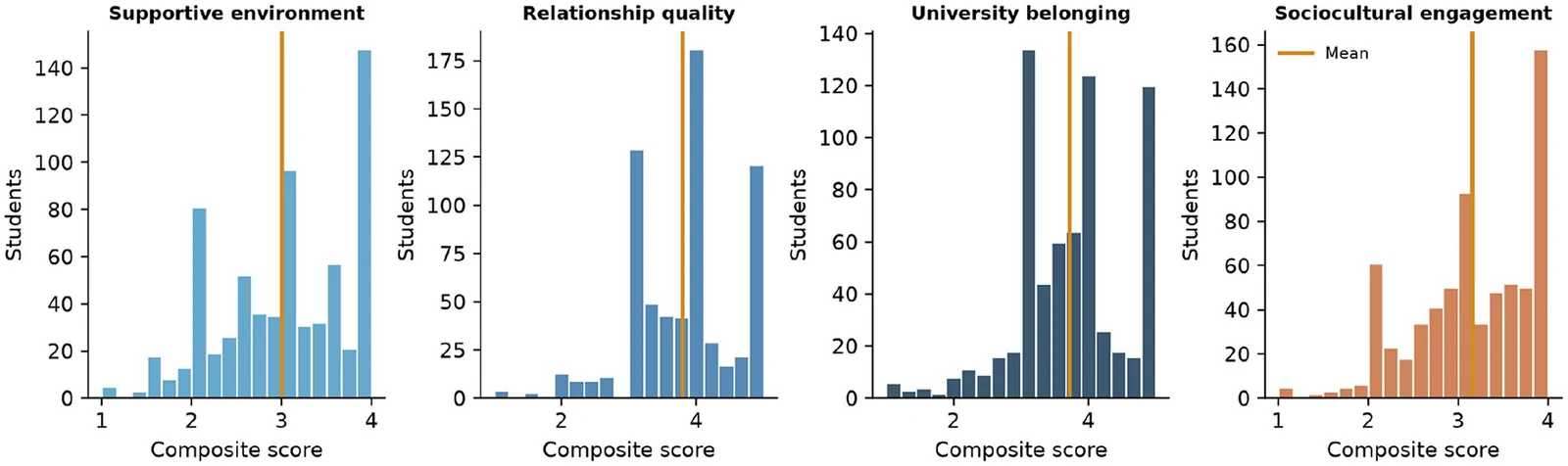
Observed distributions of the four focal composite scores.

### 3.2 Bivariate associations

All four focal constructs were positively correlated. Supportive environment and relationship quality correlated with belonging (*r* = 0.615 and *r* = 0.588, respectively) and with sociocultural engagement (*r* = 0.426 and *r* = 0.448). Belonging and sociocultural engagement were moderately associated (*r* = 0.378). The full matrix appears in Table 3.

**Table 3:** Bivariate correlations among the focal constructs (*N* = 683).

Variable	1	2	3	4
1. Supportive environment	–	–	–	–
2. Relationship quality	0.565	–	–	–
3. University belonging	0.615	0.588	–	–
4. Sociocultural engagement	0.426	0.448	0.378	–

### 3.3 Polynomial model

The primary complete-case model included 661 students and explained 47.3% of the variance in belonging. At the centered reference point, supportive environment (*b* = 1.556, HC3 SE = 0.205, *p* < 0.001) and relationship quality (*b* = 1.603, HC3 SE = 0.293, *p* < 0.001) were positive; the squared terms and interaction were not supported. Age had a small positive robust association (*b* = 0.040, HC3 SE = 0.018, *p* = 0.030), whereas gender did not. The secondary model explained 25.6% of sociocultural engagement. Support (*b* = 0.575, HC3 SE = 0.183, *p* = 0.002) and relationships (*b* = 1.145, HC3 SE = 0.263, *p* < 0.001) were positive, and the polynomial terms were unsupported. Full HC3 results appear in Table 4.

**Table 4:** Polynomial regression coefficients with HC3 heteroskedasticity-robust inference (*n* = 661).

Outcome	Term	*b*	HC3 SE	*t*	*p*
Belonging	Intercept	3.227	0.042	77.28	< 0.001
Belonging	Support	1.556	0.205	7.60	< 0.001
Belonging	Relationships	1.603	0.293	5.46	< 0.001
Belonging	Support^2^	–0.154	0.550	–0.28	0.779
Belonging	Support × relationships	–0.404	0.952	–0.42	0.672
Belonging	Relationships^2^	–0.534	0.835	–0.64	0.523
Belonging	Age (standardized)	0.040	0.018	2.18	0.030
Belonging	Gender (centered)	–0.017	0.055	–0.31	0.753
SCE	Intercept	2.820	0.041	69.25	< 0.001
SCE	Support	0.575	0.183	3.15	0.002
SCE	Relationships	1.145	0.263	4.36	< 0.001
SCE	Support^2^	0.414	0.518	0.80	0.424
SCE	Support × relationships	–0.155	0.890	–0.17	0.862
SCE	Relationships^2^	–0.275	0.731	–0.38	0.707
SCE	Age (standardized)	–0.015	0.030	–0.50	0.617
SCE	Gender (centered)	–0.128	0.052	–2.45	0.015

SCE, sociocultural engagement.

### 3.4 Response-surface parameters

For belonging, the joint-resource slope was positive (*a*_1_ = 3.159, 95% bootstrap CI [2.596, 3.602], HC3 *p* < 0.001). The bootstrap interval did not support congruence-line curvature [*a*_2_ = −1.092, CI (–2.058, 0.225)], directional difference [*a*_3_ = −0.047, CI (–0.945, 0.738)], or mismatch curvature (*a*_4_ = −0.284, CI [–3.807, 3.304]). Although the HC3 delta-method test for *a*_2_ was marginally below 0.05, its bootstrap interval crossed zero; we therefore do not make a curvature claim. For sociocultural engagement, the joint-resource slope was likewise positive [*a*_1_ = 1.721, CI (1.233, 2.133), HC3 *p* < 0.001], while *a*_2_ = −0.016, *a*_3_ = −0.570, and *a*_4_ = 0.294 all had bootstrap intervals crossing zero. The supported pattern is a joint resource gradient for both outcomes, not a matching or imbalance effect. See Table 5 and Figure 4.

**Table 5:** Response-surface parameters with bootstrap confidence intervals and HC3 inference.

Outcome	Parameter	Estimate	95% bootstrap CI	HC3 SE	HC3 *p*
Belonging	*a*_1_ joint slope	3.159	[2.596, 3.602]	0.251	< 0.001
Belonging	*a*_2_ joint curvature	–1.092	[–2.058, 0.225]	0.552	0.048
Belonging	*a*_3_ directional difference	–0.047	[–0.945, 0.738]	0.440	0.916
Belonging	*a*_4_ mismatch curvature	–0.284	[–3.807, 3.304]	1.943	0.884
SCE	*a*_1_ joint slope	1.721	[1.233, 2.133]	0.241	< 0.001
SCE	*a*_2_ joint curvature	–0.016	[–0.967, 1.154]	0.556	0.976
SCE	*a*_3_ directional difference	–0.570	[–1.365, 0.062]	0.383	0.138
SCE	*a*_4_ mismatch curvature	0.294	[–3.286, 3.506]	1.798	0.870

SCE, sociocultural engagement.

**Figure 4:**
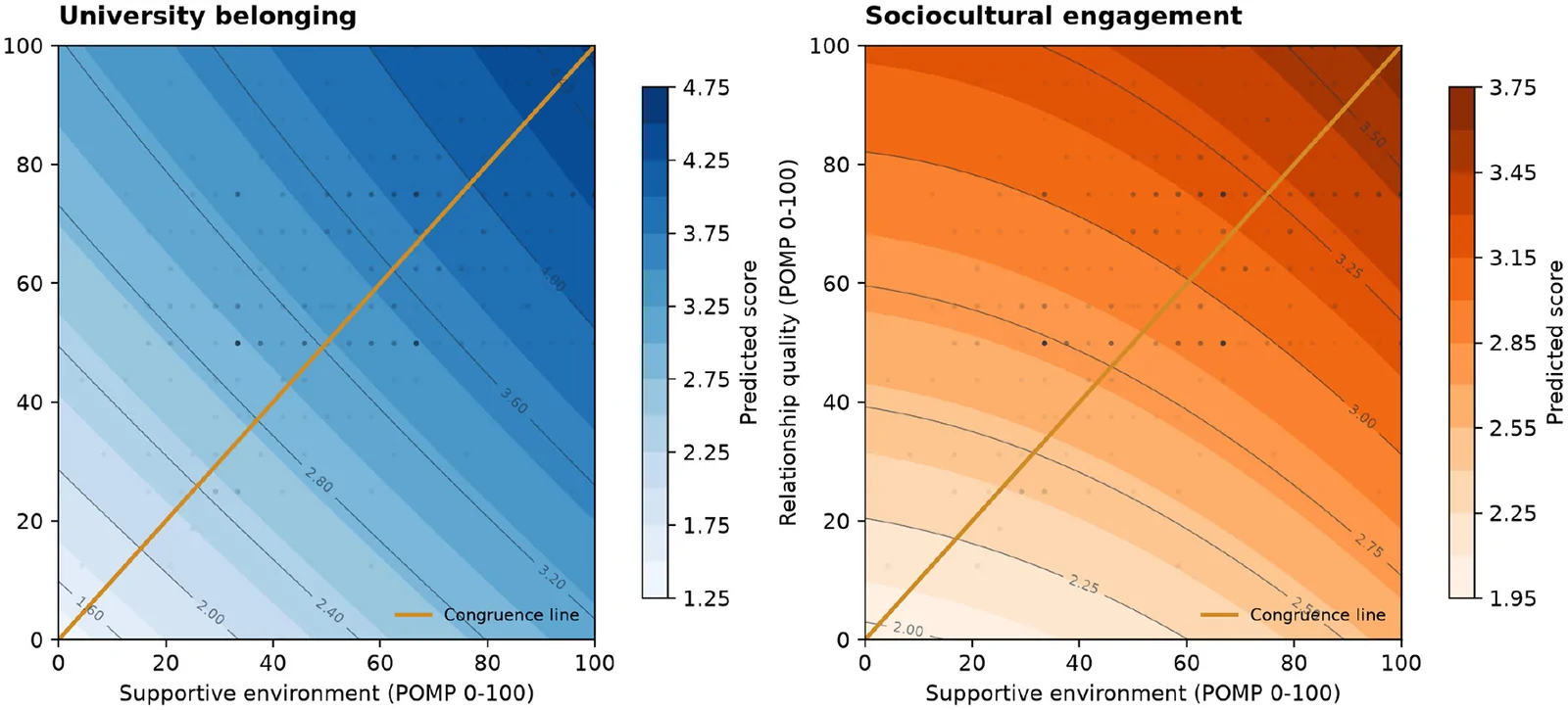
Predicted university belonging and sociocultural engagement across supportive campus environment and relationship quality.

### 3.5 Robustness and uncertainty

Removing the two exact duplicate records produced nearly identical belonging results (*n* = 659, *R*^2^ = 0.477, *a*_1_ = 3.163) and sociocultural engagement results (*R*^2^ = 0.254, *a*_1_ = 1.716). In the primary belonging model, 41 observations exceeded Cook's *D*>4/*n* (threshold 0.00605; maximum 0.150). Removing them increased belonging *R*^2^ from 0.473 to 0.506 and retained a strong positive joint slope (*a*_1_ = 2.915); it reduced the apparent negative joint curvature from *a*_2_ = −1.092 to −0.221. The corresponding sociocultural model retained a positive joint slope (*a*_1_ = 1.390). Thus the central joint-resource result is stable, whereas curvature estimates are influence-sensitive and should not be emphasized. Figure 5 and Table 6 summarize these checks.

**Figure 5:**
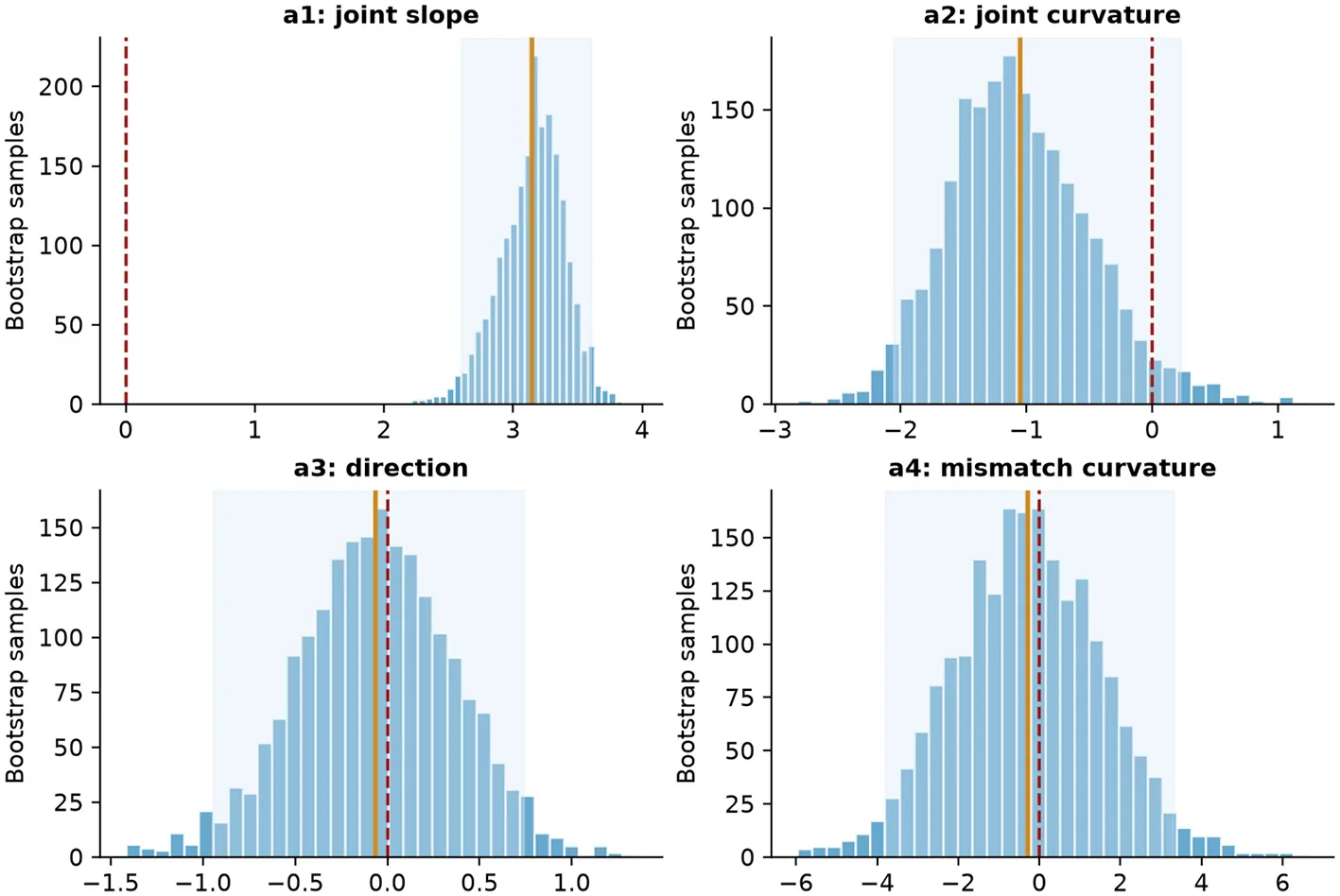
Bootstrap distributions of the four response-surface parameters.

**Table 6:** Duplicate-record and influence-point sensitivity analyses.

Outcome	Analysis	*n*	*R* ^2^	*a*_1_	*a*_2_	*a*_3_	*a*_4_
Belonging	Primary complete cases	661	0.473	3.159	–1.092	–0.047	–0.284
Belonging	Exact duplicates removed	659	0.477	3.163	–1.054	–0.055	–0.319
Belonging	Cook's *D*>4/*n* removed	620	0.506	2.915	–0.221	–0.346	–0.079
SCE	Primary complete cases	661	0.256	1.721	–0.016	–0.570	0.294
SCE	Exact duplicates removed	659	0.254	1.716	–0.029	–0.568	0.285
SCE	Cook's *D*>4/*n* removed	620	0.231	1.390	0.763	–0.947	–1.781

SCE, sociocultural engagement.

## 4 Discussion

### 4.1 A joint resource advantage, not a matching effect

The central finding is a replicated pattern across two distinct outcomes: university belonging and sociocultural engagement were higher where students simultaneously perceived a supportive campus environment and high-quality relationships. For both surfaces, the supported feature was a positive joint-resource slope. Bootstrap, HC3, duplicate-removal, and influence-point results did not justify an optimal balance, privileged equality point, directional-imbalance effect, or mismatch penalty. This result supports and extends the source study. Kassab et al. (2024a) showed positive direct paths from campus environment and relationship quality; the present RSA shows that their combined surface is best described as a cumulative resource gradient, not a special benefit of equality between the two resources. The evidence therefore supports resource accumulation rather than congruence.

This distinction matters for both theory and practice. A matching interpretation would imply that equal levels are valuable even when both are low, or that imbalance is harmful beyond the absolute resource levels. The observed surface does not justify either claim. Instead, institutional and interpersonal resources appear to contribute in parallel. This conclusion is consistent with systematic evidence that belonging is responsive to both university-wide practices and interpersonal experiences (Allen et al., 2024), and with the recent meta-analytic association of university belonging with academic and wellbeing outcomes (van Kessel et al., 2025). It also complicates any treatment of belonging as a single undifferentiated experience: recent profile research separates social and academic belonging and shows that they relate differently to academic and psychological outcomes (Lawrie et al., 2025). Our measures are broader and cannot reproduce those profiles, but the same caution applies—institutions should not assume that one favorable domain stands in for all others. A university with strong services but weak everyday relationships may still provide some basis for belonging; supportive peers and faculty may likewise matter when institutional support is imperfect. However, the highest predicted belonging occurred when both layers were favorable.

### 4.2 Belonging as a relational campus-identity outcome

The findings are consistent with a relational account of campus identity. Students do not infer membership from formal enrollment alone. They encounter institutional priorities through services, events, support practices, and opportunities for cross-background contact, and they encounter the social meaning of membership through peers, faculty, advisors, and staff. Belonging can thus be interpreted as a judgment that the institution and its relationships make a place for the student. This interpretation resonates with communities-of-practice theory, in which identity develops through trajectories of participation and recognition (Wenger, 1998, 2010). It is also compatible with Covarrubias (2024)'s argument that university cultural scripts make acceptance both personal and political. The present aggregate measures support the importance of institutional and relational conditions, but they cannot determine whose identities are affirmed, assimilated, or marginalized by particular practices.

At the same time, the evidence is only one slice of co-construction. Mutual constitution implies ongoing reciprocal processes: students interpret, reproduce, resist, and alter institutional practices, while institutions shape the opportunities and meanings available to them. A cross-sectional survey captures students' perceptions at one point, not those reciprocal sequences. The study therefore contributes to the Research Topic by quantifying relational conditions associated with campus-based identity, while leaving dynamic identity work for longitudinal, narrative, observational, or experimental research.

The culturally diverse UAE context strengthens the relevance of this bounded interpretation without supporting overgeneralization. The sociocultural-engagement items directly capture interaction across cultural, ethnic, religious, and economic difference, perspective taking, communication adaptation, and learning about oneself through others. That makes the secondary outcome more proximal to multicultural participation than belonging alone. It still does not measure heritage-culture orientation, bicultural integration, migration status, language identity, or reciprocal change in institutional culture. The appropriate inference concerns conditions associated with participation and belonging in a diverse campus, not international-student acculturation or identity co-construction as an observed process.

### 4.3 Methodological contribution

The reanalysis illustrates what RSA adds to an established linear model. Rather than estimating only net paths, it asks how belonging and sociocultural engagement vary across combinations of institutional and interpersonal resources. Reporting all four surface parameters, bootstrap intervals, HC3 inference, and influence sensitivity separates a well-supported joint gradient from unsupported or unstable curvature and mismatch narratives. Those negative results are part of the contribution because they narrow the claims the data can sustain.

The transparent workflow is equally important for open-data secondary research. Repository datasets can enable new questions, but reuse does not eliminate the need for provenance checks. Here, the 683/638 discrepancy could have been silently ignored or forced to match the article. Reporting the released file, tracing internal consistency clues, and preserving the discrepancy makes the inferential chain auditable. This practice is particularly important when a secondary analysis seeks publication as Original Research rather than as a direct replication.

### 4.4 Practical implications

For universities, the results support a concrete two-layer diagnostic rather than a generic call to “increase belonging.” A program can administer a brief, repeated pulse survey of perceived campus support and relationship quality, inspect the two scores separately, and choose the weaker layer as the first operational target while continuing to monitor both. Consider an illustrative health-professions cohort that reports easy access to tutoring, wellbeing services, and student activities but low relationship quality. The evidence would not justify creating another service; a more aligned response would be stable faculty-adviser assignments, scheduled small-group check-ins, peer mentoring across cohorts, and faculty development on respectful feedback. Conversely, a cohort with strong peer and faculty ties but low perceived campus support would call for clearer service navigation, protected time to use support, rapid referral pathways, and removal of administrative access barriers. Because no mismatch penalty was detected, either layer can be improved first; because the joint slope was positive, progress should ultimately be tracked across both.

For health-professions programs, a feasible paired initiative could combine institutional infrastructure with repeated relational contact. For example, an incoming cohort could receive a named service navigator and a persistent faculty adviser, work in culturally mixed peer teams on clinical cases, and complete structured check-ins at weeks 2, 6, and 12. Implementation indicators could include adviser continuity, referral completion, participation in cross-cultural activities, and response time to student concerns; belonging and sociocultural engagement could be reassessed at the same intervals. Units should also disaggregate these indicators where ethically and statistically defensible, because aggregate improvement can conceal unequal experiences of acceptance. Such a program would connect institutional commitments to the psychologically safe relationships through which students learn membership, voice, and collaboration (Tsuei et al., 2019; Covarrubias, 2024). These examples are hypotheses for prospective evaluation, not interventions proven by the present cross-sectional data, and they should be tested with comparison groups or interrupted time-series designs. This interpretation is consistent with research on professional identity formation (Burkhardt et al., 2015; Mukhalalati et al., 2024).

### 4.5 Limitations and future research

First, the cross-sectional design cannot establish causal direction, temporal order, or reciprocal co-construction. Students with stronger belonging may evaluate their environment and relationships more positively. Longitudinal cross-lagged, intensive repeated-measures, or experimental designs are needed to distinguish selection, perception, and contextual influence. Second, all focal variables were self-reported in one survey, creating common-method, consistency-motif, and evaluative-response risks. The released survey contains neither a marker variable nor an independent measurement source, so the size of common-method bias cannot be estimated from these data. Future studies should separate predictor and outcome measurement in time and combine perceptions with administrative indicators, behavioral participation, network data, and observations of institutional practice.

Third, the convenience sample came from one health-professions university in the United Arab Emirates, with an overrepresentation of women and students from Asian and Middle Eastern backgrounds in the original report. The single-institution design provides no institution-level variation, and the released file lacks sufficiently detailed ethnicity, nationality, socioeconomic position, migration history, language, disability, and gender-identity measures. Consequently, neither multilevel institutional effects nor intersectional subgroup surfaces can be estimated responsibly. The result should be tested across institutions, disciplines, national settings, and marginalized student groups using probability-based or deliberately stratified recruitment where feasible. Multi-group or multilevel models could then examine whether the response surface differs across social positions, but such analyses require sufficiently measured and powered groups rather than *post hoc* claims of intersectionality.

Fourth, complete-case analysis assumes that excluded records do not differ in ways that materially change the surface. Missingness was limited, but multiple imputation and missingness-pattern sensitivity would strengthen future work. Fifth, the score distributions were concentrated toward higher resource levels, making the extreme low-low region less data-dense. Sixth, the binary gender code in the released data is inadequate for contemporary identity-sensitive analysis and was used only as a limited covariate. Seventh, the secondary analysis was not preregistered, so its questions should be treated as exploratory-confirmatory rather than prospectively confirmatory. The missing-data assumptions therefore remain consequential (Roth, 1994).

Finally, the repository and linked article report different source sample counts (683 and 638, respectively). This reanalysis treats the released Version 1 file as the explicit analysis object, reports its 683 records, and bases inference on the documented 661-case intersection. No exclusion variable or reproducible rule in the public materials yields 638. The discrepancy therefore prevents full reconciliation with the source report, but it does not create ambiguity about which observations entered the present models. Independent reproduction should use Version 1 of the repository and the complete-case rules reported above; clarification from the data creators would still strengthen provenance confidence.

## 5 Conclusion

University belonging and sociocultural engagement were both higher where students perceived stronger institutional support and higher-quality relationships. HC3 inference, duplicate removal, and influence-point sensitivity retained the positive joint-resource pattern, whereas no stable matching, directional-imbalance, or mismatch-curvature effect emerged. The results provide a bounded quantitative contribution to the Research Topic: culturally diverse campus participation and belonging are associated with institutional and relational conditions, but the cross-sectional measures do not directly observe identity co-construction. Longitudinal and multi-institutional work is needed to test how students and institutional cultures shape one another over time.

## Data Availability

Publicly available datasets were analyzed in this study. The existing dataset analyzed in this study is publicly available in Mendeley Data (Version 1; Kassab et al., 2024b). The dataset is licensed under CC BY 4.0.

## Supplementary material

SUPPLEMENTARY DATA SHEET 1 Reproducibility package containing Python analysis scripts, dependency specifications, source-data access instructions, and reference numerical outputs for the polynomial regression and response-surface analyses, including bootstrap inference, HC3-robust inference, duplicate-record checks, and Cook's D sensitivity analyses.
